# Image Retrieval Method for Multiscale Objects from Optical Colonoscopy Images

**DOI:** 10.1155/2017/7089213

**Published:** 2017-02-01

**Authors:** Hirokazu Nosato, Hidenori Sakanashi, Eiichi Takahashi, Masahiro Murakawa, Hiroshi Aoki, Ken Takeuchi, Yasuo Suzuki

**Affiliations:** ^1^National Institute of Advanced Industrial Science and Technology (AIST), Artificial Intelligence Research Center (AIRC), 1-1-1 Umezono, Tsukuba, Ibaraki 305-8560, Japan; ^2^Toho University Sakura Medical Center, 564-1 Shimoshizu, Sakura, Chiba 285-8741, Japan

## Abstract

Optical colonoscopy is the most common approach to diagnosing bowel diseases through direct colon and rectum inspections. Periodic optical colonoscopy examinations are particularly important for detecting cancers at early stages while still treatable. However, diagnostic accuracy is highly dependent on both the experience and knowledge of the medical doctor. Moreover, it is extremely difficult, even for specialist doctors, to detect the early stages of cancer when obscured by inflammations of the colonic mucosa due to intractable inflammatory bowel diseases, such as ulcerative colitis. Thus, to assist the UC diagnosis, it is necessary to develop a new technology that can retrieve similar cases of diagnostic target image from cases in the past that stored the diagnosed images with various symptoms of colonic mucosa. In order to assist diagnoses with optical colonoscopy, this paper proposes a retrieval method for colonoscopy images that can cope with multiscale objects. The proposed method can retrieve similar colonoscopy images despite varying visible sizes of the target objects. Through three experiments conducted with real clinical colonoscopy images, we demonstrate that the method is able to retrieve objects of any visible size and any location at a high level of accuracy.

## 1. Introduction

Recently, the number of cancer patients around the world has been increasing. Within recent decades, the number of newly diagnosed patients has increased by 420,000 from 1992, reaching approximately 870,000 cases within Japan in 2012 [1]. Gastroenterological cancers, which include stomach, colon, and rectum areas, have particularly high incidence rates, and are projected to become the most common cause of death by 2014 [2]. On the other hand, data also indicates that there have been significant improvements in survival rates at 5 years after diagnosis, because recent medical advances are increasingly helping to detect cancer at treatable early stages of development. For cancers of the colon and rectum areas, optical colonoscopy is the most popular approach towards screening, surveillance, and treatment. Using colonoscopy, medical doctors can directly observe the colonic mucosa and obtain detailed information about the condition of the colon. This means that optical colonoscopy is especially suited for the early detection of cancer.

However, diagnostic accuracy is highly dependent on both the experience and knowledge of medical doctors, because they must make immediate identifications by closely observing the microscopy images while simultaneously carrying out the colonoscopy examination. It is extremely difficult, even for specialist doctors, to detect the early stages of a cancer within inflammations of the colonic mucosa due to intractable inflammatory bowel diseases (IBDs), such as ulcerative colitis (UC) and Crohn's disease (CD), which result in a wide variety in the appearances of colonic mucosa associated with IBDs symptoms. In particular, UC has a number of serious symptoms, such as intermittent bloody diarrhea, rectal urgency, and tenesmus [3], which dramatically affect the quality of life (QOL) of UC patients [4]. Moreover, prolonged active colonic inflammations increase the risks of colorectal cancer with incidences at approximately 3.7% on average and between 2% and 19% from 10 to 30 years after diagnosis [5]. Thus, periodic examinations using optical colonoscopy are most important to understand exactly how IBDs advance to maintain patient QOL and to prevent the onset of colorectal cancer.

In order to reduce mortalities due to colorectal cancer associated with IBDs, the standard recommendations for early detection in the United States and Europe are to conduct surveillance colonoscopy with random biopsies, where multiple tissue samples are taken at equal intervals across the broad areas of flat and apparently abnormal colonic mucosa [6]. In contrast, the Japanese guideline for the management of UC suggests targeted biopsies, where tissue samples are only taken from locations suspected of being cancerous, rather than random biopsies [7]. Since targeted biopsies are regarded as being less painful for patients compared to random biopsies, it is vital to develop assistance technology that can positively contribute to improving detection accuracies for targeted biopsies.

Seeking to enhance UC diagnoses using optical colonoscopy, we have already proposed a method of content-based image retrieval [8], which was developed based on image-recognition techniques, such as image preprocessing and geometrical feature extraction. This previous retrieval method can return reference images based on degree of similarity to the images being diagnosed and can potentially furnish the medical records associated with the retrieved images to assist the UC diagnosis. However, this method is unable to adequately deal with variations in the visible sizes of retrieved objects within optical colonoscopy images. If colonoscopy images are captured with a discrete shooting distance, the spatial resolutions related to target mucosal surface are different. Consequently, the visible size of a target object within the colonoscopy images will be different and influence the process of feature extraction, which, in turn, will affect retrieval results. Moreover, it is even possible to obtain different results for the same target object, due to differences in the visible sizes of the object within different images. Thus, to realize effective assistance technology for practical applications, it is essential to have a new retrieval method that is not totally dependent on the visible dimensions.

Compensating for the effects of visible sizes within optical colonoscopy images, this study seeks to realize a method of retrieving multiscale objects from optical colonoscopy images based on higher-order local autocorrelation (HLAC) [9]. In order to retrieve similar objects that differ in their visible sizes, the proposed method generates integral HLAC feature tables that are calculated by a newly extended HLAC extraction method based on an integral image technique [10]. As the HLAC features for an arbitrarily sized clipping area can be reconfigured from a table of integral HLAC features, this approach is far more efficient than the conventional HLAC method. Preliminary results for this work have been reported and displayed in BioCAS 2015 [11]. This paper provides more detailed descriptions of the proposed method, the experimental setup, and results including the additional experiment to demonstrate the potential of the methodology for multiscale retrieving using actual colonoscopy images with low computational cost.

This paper is organized as follows: Section 2 outlines the basic approach of the proposed retrieving method.  Section 3 explains the details of the methodology for retrieving multiscale objects.  Section 4 presents the results of three experiments conducted to verify the proposed method. Finally, a summary of the study is provided in Section 5.

## 2. Basic Procedure of the Proposed Retrieving Method

### 2.1. Overview

**Figure 1:**
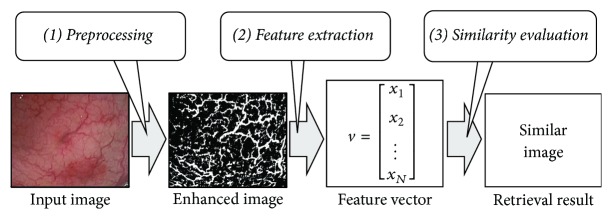
Overview of the basic retrieval procedure.

The proposed method is essentially an extension of our previous content-based image retrieval (CBIR) method [8], which effectively combines three key phases of preprocessing, geometric feature extraction, and similarity evaluation, as shown in Figure 1. This previous retrieval method based on geometric features, which represent visual textures and appearances of mucosal symptoms, can retrieve similar colonoscopy images close to the evaluation by human sight. In the preprocessing phase, some characteristics of colonoscopy images are emphasized with an appropriate color space converted from the RGB color space of the original colonoscopy image with the RGB color model to capture the condition of colonic mucosa. In the feature extraction phase, the HLAC method, as a common recognition technique, extracts the geometrical features that can be regarded as characteristics for the overall appearance of the colonic mucosa from the preprocessed colonoscopy images. In the similarity evaluation phase, in order to retrieve images similar to an input image, similarity evaluations compare the feature vector of an input image with every stored target feature vector within a previously prepared database. Then, as a retrieval result, the similar image with a feature vector that yields the largest similarity value is selected. The following subsections describe the basic procedures of these key phases based on our previous work [8].

### 2.2. Image Processing

Some studies have reported that vascular patterns and other aspects can be better distinguished from the green element within the RGB color space than from the red and blue elements in classifying the severity of colonic mucosa [12, 13]. However, the RGB color space is affected by the light-dark contrasts within actual colonoscopy images that are dependent on the capturing conditions, such as illumination, shooting distance (colonoscopy-to-subject distance), and angle. Accordingly, it is important to reduce the effects of light-dark contrasts to realize image retrieval for colonoscopy images.

**Figure 2:**
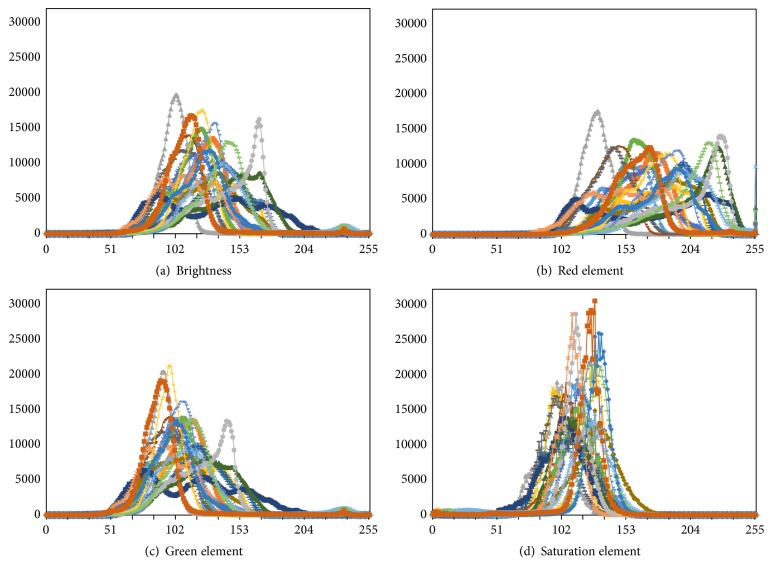
Histograms of the color elements.

**Figure 3:**
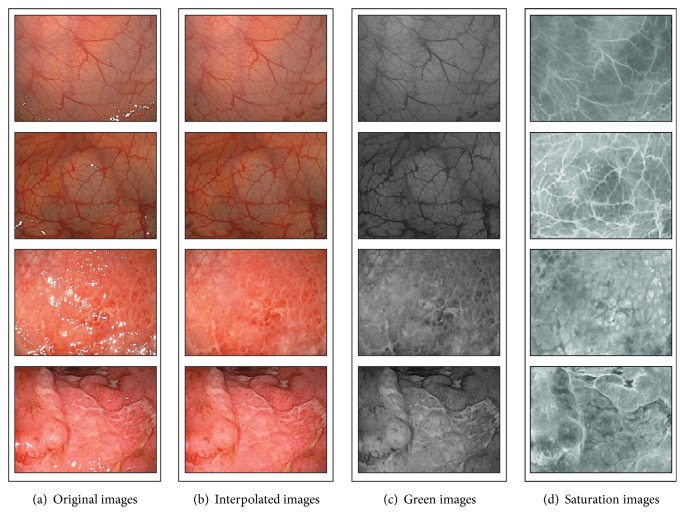
The preprocessed colonoscopy images.

In our previous work [14], we reported that the saturation element of the HSV color space results in robust histograms for images of uneven brightness.  Figure 2 shows the histograms of 20 normal colonoscopy images taken under various illumination conditions for different color elements of the RGB and HSV color spaces. As presented in Figure 2(d), the histogram for the saturation element has a sharper distribution than the histograms for the other elements. Moreover, the saturation element can express the appearance of red-based colored colonic mucosa [14]. Accordingly, for preprocessing, we adopt the saturation element converted from the original colonoscopy images for the RGB color space to reduce the effects of light-dark contrasts and to enhance the appearances of colonic mucosae. In addition, bright blobs within colonic images are interpolated by a method of image inpainting [15] prior to conversion, because the saturation element cannot be converted from bright blobs, as shown in Figure 3.

### 2.3. Feature Extraction

Some CBIR methods have been developed as support technology for medical images [16]. In addition to basic color features, texture and shape features are usually used in CBIR for medical images. However, because texture and shape features must be extracted through complex analyses with high computational costs, they are unsuitable for optical colonoscopy examinations that require quick feedback. In order to achieve feature extraction at low computational costs, the higher-order local autocorrelation (HLAC) method [9] was adopted as the basic framework of the feature extraction for CBIR in previous work [8]. The HLAC method can extract geometrical features, such as area, contour, and shape of objects, from an input image according to local autocorrelations of pixels. The HLAC features, which have advantages such as a shift-invariance and a frame-additivity, represent the expressed characteristics for a whole image, derived from the product-sum operations of the following formula that represents autocorrelations:

(1) $$\begin{array}{c} F_{N}=\underset{r}{\sum }I\left(\;r\;\right)\cdot I\left(\;r+a_{1}\;\right)\cdot \ldots \cdot I\left(\;r+a_{N}\;\right), \end{array}$$

where *N* is the order of autocorrelations, *r* is the* X*-*Y* coordinate vector of the colonoscopy image, *I*(*r*) is the pixel value at *r*, and *a*_*i*_  (*i* = 1 ⋯ *M*) are displacement vectors around *r*. For general HLAC of gray-scaled images, taking into consideration the shift-invariant property, 35 masks are utilized that are formed by the configurations of *r* and *a*_*i*_, restricted by the second orders of autocorrelation (*N* = 0,1, 2) within a 3 × 3 area around *r*.

**Figure 4:**
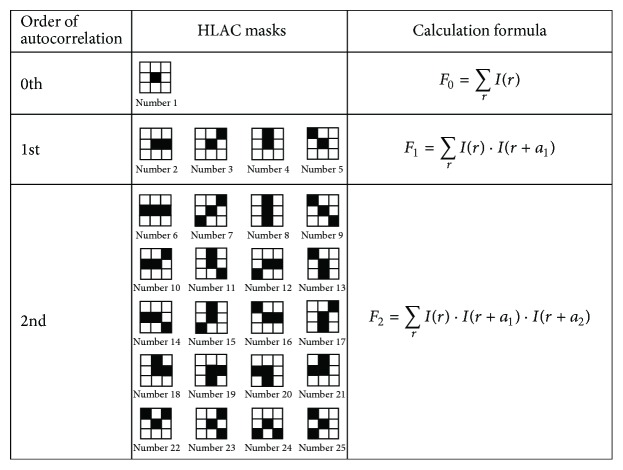
HLAC masks up to second-order autocorrelation for the proposed retrieving method.

In this paper, to enhance the effectiveness of autocorrelations between neighborhood pixels, 25 HLAC masks, excluding the 10 HLAC masks with square and cube calculations at* r*, are adopted for the proposed retrieving method, as shown in Figure 4. At each autocorrelation order, the HLAC features geometrically represent the sum total of the brightness (number 1), the directions (number 2 through number 5), and linear and curve lines (number 6 through number 25), respectively. In each mask, the black cells indicate the target pixels used for multiplication while the white cells indicate unused pixels. Each mask scans the whole image and the sums of products are calculated for the matched target pixels. The calculated sums for all masks are concatenated to form a 25-dimensional vector,* v*, as the HLAC features that are regarded as a property of the input image.

### 2.4. Similarity Evaluation

To evaluate the similarity between a query object and any objects within the database, a similarity value is calculated from the Euclidean distance between both features. Euclidean distance is adopted in this study, because it is a simple way to calculate a similarity value between vectors at low computational costs. Moreover, for multiscale objects, the retrieval task increases exponentially in complexity as a function of the variety in the sizes and locations of arbitrary areas within the target images. Accordingly, to reduce the number of comparisons, two selection methods are adopted in combination with this similarity evaluation, as described in Section 3.3.

## 3. Details of the Methodology for Retrieving Multiscale Objects

### 3.1. Basic Idea

For retrieving multiscale objects, the feature extraction procedure for all retrieval candidates, which are clipped with arbitrary area from a target image to include various visible sizes of the target object, is required. The conventional HLAC feature extraction takes a high computational cost to repetitively compute HLAC features for all retrieval candidates. Therefore, this study significantly enhances the HLAC feature extraction component in order to handle the retrieval of multiscale objects by utilizing the integral image technique [10] to reduce the computational costs.

**Figure 5:**
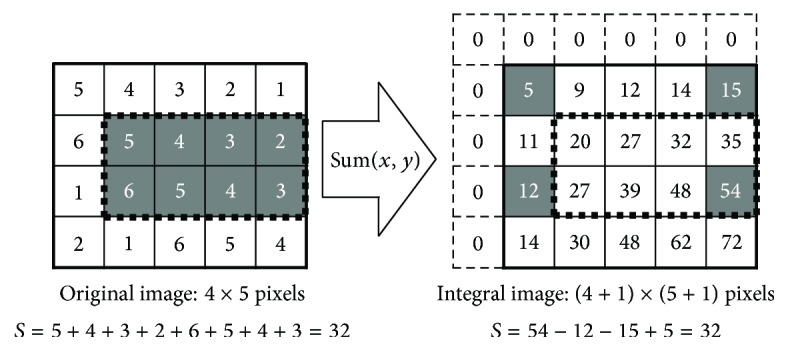
Pixel configurations for the original image and its integral image.

Figure 5 illustrates pixel configurations for an original image and its integral image. Each pixel value of the integral image is calculated as the total sum of the pixel values within a rectangular region that extends from the origin point to the corresponding pixel of the original image. Thus, it can be achieved simply by executing addition or subtraction operations four times using the pixel values of the integral image to calculate a sum total of the arbitrary area (the rectangular area) of the original image irrespective of the scale of the target area, as illustrated by the sample calculations for the rectangle of dotted lines shown in Figure 5. This calculation algorithm of sum has a high affinity to the additivity and can simplify the HLAC feature calculation for a clipped area.

**Figure 6:**
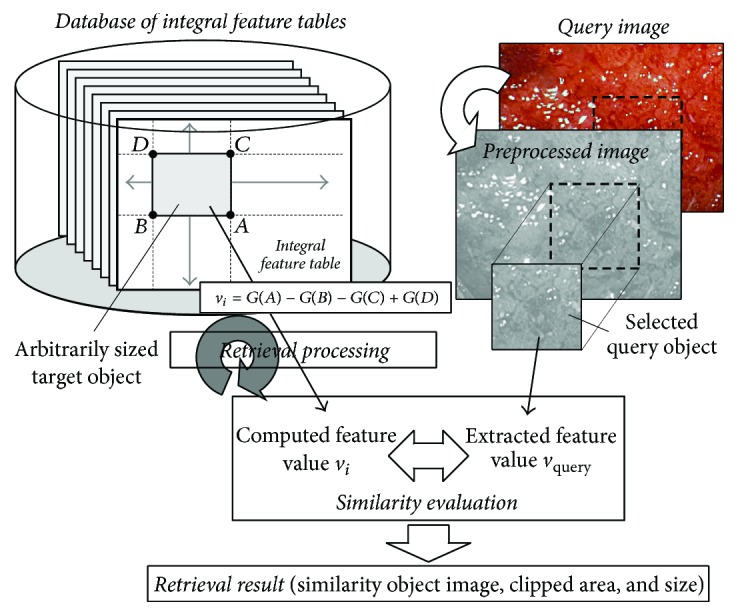
Illustration of the proposed retrieval method using the integral feature table.

In this paper, by applying this integral technique to a database of extracted HLAC features for retrieval candidates, it is possible to efficiently compute the features of an arbitrarily sized region so that objects of various visible sizes can be retrieved during retrieval processing, as shown in Figure 6. A region, which is arbitrarily determined both in terms of its size and location, is clipped from the integral feature table and a similarity evaluation between its computed feature and the feature of an extracted query is calculated. Retrieval processing is repetitively executed for all retrieval candidates within the database and the results of the similarity evaluations are compared in producing the retrieval results. The main procedures of proposed method are described in more detail in the following subsections.

### 3.2. Integral Feature Table

**Figure 7:**
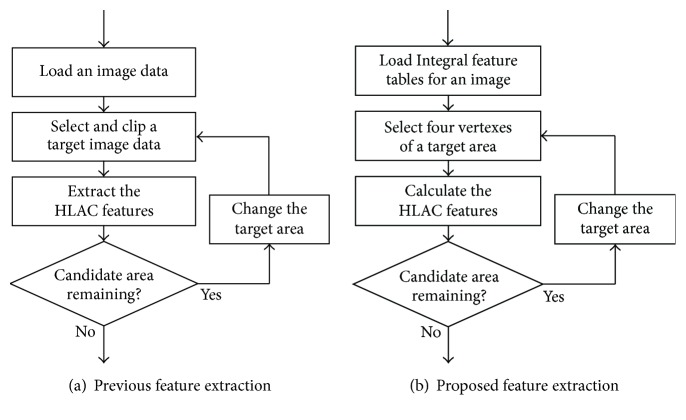
Flowcharts of the HLAC features extraction with/without the integral feature tables for the retrieval candidates.

As it is necessary to reiterate the processes of image clipping at arbitrarily determined sizes and locations including any visible size of the target object and of feature extraction in order to realize multiscale object retrieval, our previous HLAC algorithm is not particularly suitable, because the features are irreversible values and new extractions must be executed whenever the target area is changed, as shown in Figure 7(a). Accordingly, within this study, the HLAC method is extended with the incorporation of an integral feature table that can easily generate the feature of an arbitrary area at low computational costs based on integral image techniques [10]. The integral feature value *G* at any* X*-*Y* coordinate *r*(*x*, *y*) in the integral feature table is the sum of all the autocorrelations *f* at* r*, according to the following formula:

(2) $$\begin{array}{c} G\left(\;r\left(\;x,y\;\right)\;\right)=\underset{x^{\prime }\leq x}{\sum }\;\underset{y^{\prime }\leq y}{\sum }f\left(\;r\left(\;x^{\prime },y^{\prime }\;\right)\;\right). \end{array}$$

**Figure 8:**
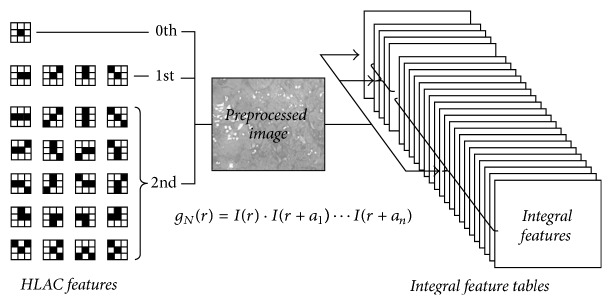
Integral feature extracting using HLAC masks.

In the database-creation phase, the integral features are generated at every pixel of a preprocessed image for every HLAC feature, according to the following formula:

(3) GNrx,y=∑x′≤x ∑y′≤yIrx′,y′·Irx′,y′+a1⋯Irx′,y′+aN

to form an integral feature table for every HLAC feature, as shown in Figure 8. Then, according to the flowchart in Figure 7(b), it is possible to compute the HLAC features,* F*, for the arbitrary region of a rectangle with the points* A*,* B*,* C,* and* D* as vertexes, as shown in the illustration of the integral feature table in Figure 6, from the integral feature value,* G*, with a light processing load according to the following formula:

(4) $$\begin{array}{c} F_{N}=G_{N}\left(\;A\;\right)-G_{N}\left(\;B\;\right)-G_{N}\left(\;C\;\right)+G_{N}\left(\;D\;\right). \end{array}$$

Furthermore, the normalized HLAC features, *F*′, indicating ratios of the computed feature values to the number of the pixels,* n*, of each arbitrary region are calculated by the following formula:

(5) $$\begin{array}{c} F_{N}^{\prime }=\frac{F_{N}}{n}. \end{array}$$

If the sizes of a target image and a query object are 400 × 300 pixels and 100 × 100 pixels, respectively, 1,271,291 candidates were clipped to be area of plus/minus 10% (from 90 × 90 pixels to 110 × 110 pixels) of the query object size while shifting them by 1 pixel in the target image. Then, in the preliminary experiment, the computing times with/without the integral feature tables were 406 msec and 417,106 msec to extract the HLAC features from all candidates, respectively. The total computing time was 1,031 msec even when including the computing time of creating the integral feature tables from a target image. Thus, this proposed integral feature table can easily change the sizes and locations of all retrieval candidates with a low computational cost to realize multiscale retrieval.

### 3.3. Retrieval Processing

In the human retrieval, the overall brightness of an image is firstly used to narrow down the candidates. After then, more detailed information are used to search the similar objects. Therefore, in this study, retrieval processing consists of the two procedures of area selection and image selection to efficiently retrieve similar objects from many retrieval targets using the 0th order HLAC feature which represents the overall brightness of an image.

**Figure 9:**
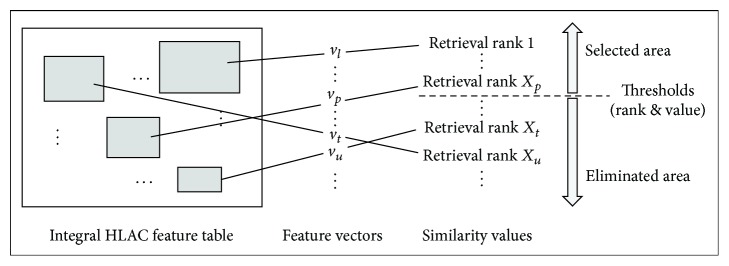
Area selection from the integral feature table.

For the area selection procedure, some areas in a target image with a high degree of similarity are selected according to the retrieval thresholds using an integral feature table at the 0th order HLAC, as shown in Figure 9. Thus, this method of area selection drastically reduces the candidates from among numerous retrieval targets during the initial step of the retrieval process. If the sizes of a target image and a query object are 400 × 300 pixels and 100 × 100 pixels, 1,271,291 candidates were clipped, as mentioned in the previous subsection. Thus, it is essential to filter down the candidates based on the area selection step prior to retrieval processing that use the detailed characteristics that are expressed by 1st- and 2nd-order HLAC features.

**Figure 10:**
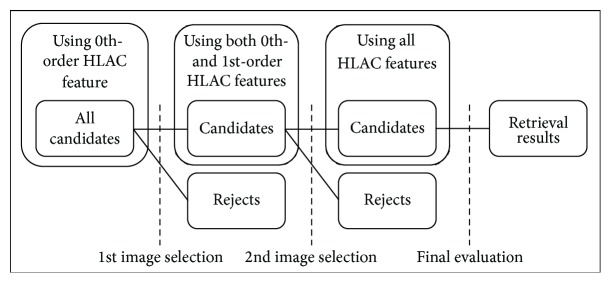
Stepwise image selection for all candidate images.

For image selection, we adopt stepwise selection for all autocorrelation orders, as shown in Figure 10. This selection can reduce the number of candidates of target images incrementally and can evaluate similarities using higher-order HLAC features step by step. The retrieval thresholds for each selection are defined separately. This stepwise algorithm can also retrieve a similar object from many retrieval targets by filtering the candidates. Accordingly, the proposed method for retrieval processing can efficiently retrieve a similar object using the brightness information represented by the 0th-order HLAC feature during the first step and more detailed information, such as a texture pattern and an object contour, is represented by the combination of higher-order HLAC features thereafter with a narrowing down of the number of candidates, just like human retrieval that proceeds by utilizing more detailed information.

## 4. Experiments

### 4.1. Experimental Conditions

This study reports on three verification experiments conducted to examine both the potential to retrieve multiscale objects and retrieval performance for actual colonoscopy images. In these experiments, 112 colonoscopy images in total, which were obtained from 13 UC patients, were used as retrieval target images and query images. The patients signed consent forms and this study was approved by the Ethics Committee of the Faculty of Medicine, Toho University, and the Committee for Ergonomic Experiments in the National Institute of Advanced Industrial Science and Technology (AIST).

Table 1 shows the experimental conditions for these verification experiments. All of retrieval target images were created by preliminary trimming of the original colonoscopy images to 800 × 600 pixels in size. In these experiments, for retrieving multiscale objects, retrieval candidates were clipped to be areas of plus/minus 10% of the query object size while shifting them by 10 pixels in the integral feature table. The three thresholds for area selection and for image selection were determined on the basis of results from prior experiments, as shown in Table 1. The details of each of the experimental data and experimental results are described in the following subsections.

### 4.2. Experiment for Capability to Retrieve Multiscale Object

**Figure 11:**
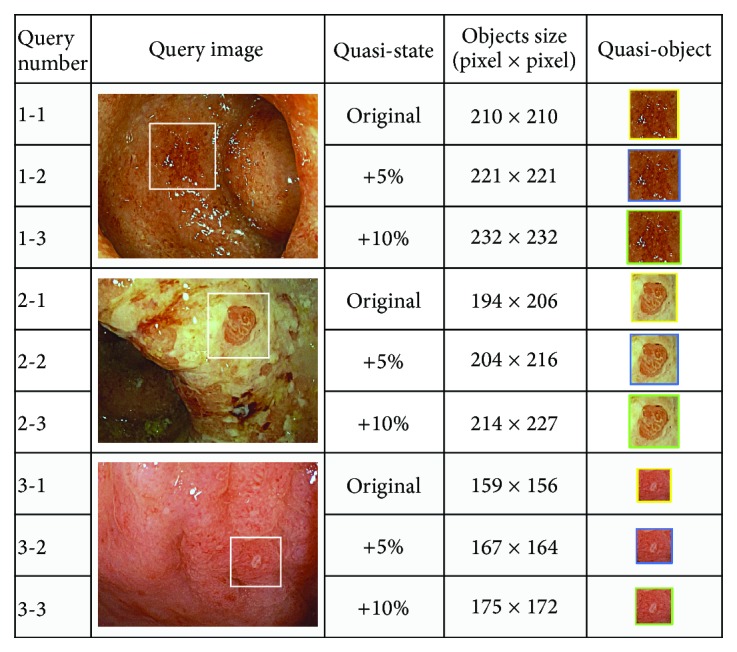
Query quasi-objects for three colonoscopy images.

This experiment was executed to inspect the performance of the proposed method in retrieving multiscale objects using quasi-images. The nine query objects for the three UC types were quasi-images that had been expanded as a clipped query object from three colonoscopy images, respectively, as shown in Figure 11. These quasi-images were regarded as different images of the same object captured with a discrete shooting distance. Thus, these quasi-images represent the multiscale objects. These three colonoscopy images were also used as target images.

**Figure 12:**
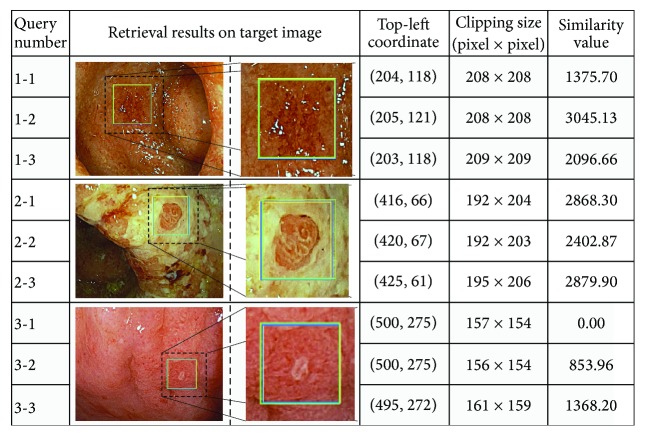
Retrieval results for query quasi-objects.

Figure 12 shows the retrieval results, which include visible result, location, clipped size, and similarity value. Comparing the clipping sizes with the object sizes in the original query objects (Figure 11), three clipped sizes for each query image are approximately the same as the original query object, which is regarded as being the correct retrieval result in terms of size. Moreover, the visible results for the target images, as shown in Figure 12, indicate that the locations of all clipped areas in the results for each query image are also approximately the same as the original query object. Thus, comparing the results for the quasi-images indicates that the proposed method is capable of retrieving objects of any size at high accuracy.

### 4.3. Experiment for Different Images of the Same Mucosal Symptom

**Figure 13:**
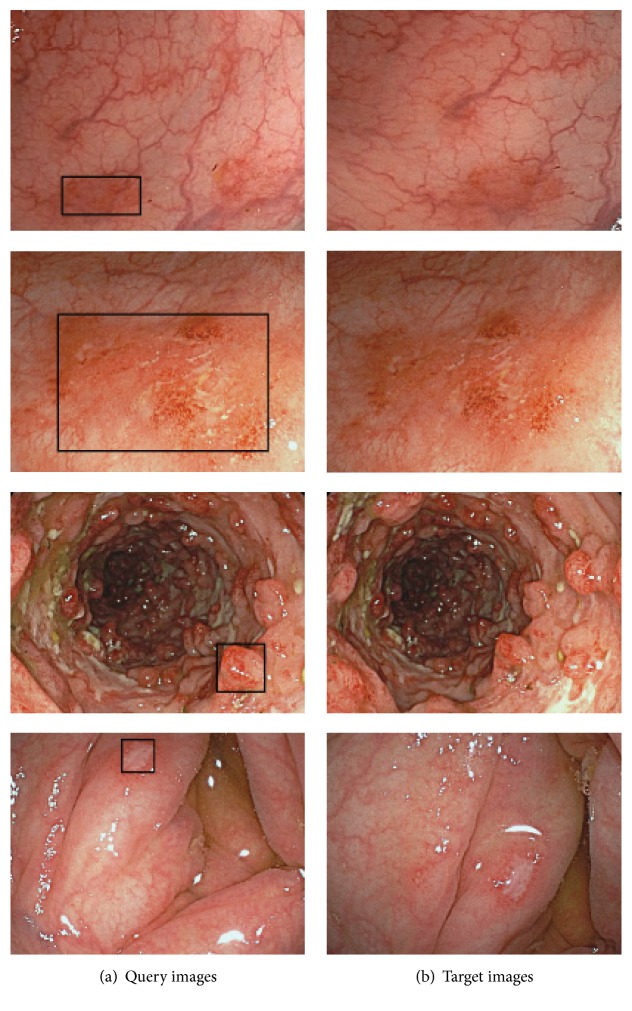
Four pairs of different images for the same mucosal symptom.

This experiment was executed to establish the performance of the proposed method in retrieving multiscale objects using different images of the same mucosal symptom. The eight colonoscopy images for four UC types were used, which were composed of four pairs of query images and target images, where the same mucosal symptom appeared at different visible sizes and at different locations, as shown in Figure 13. In this experiment, the similarity object was retrieved from the target image (Figure 13(b)) for the query object (black rectangles in Figure 13(a)), respectively.

**Figure 14:**
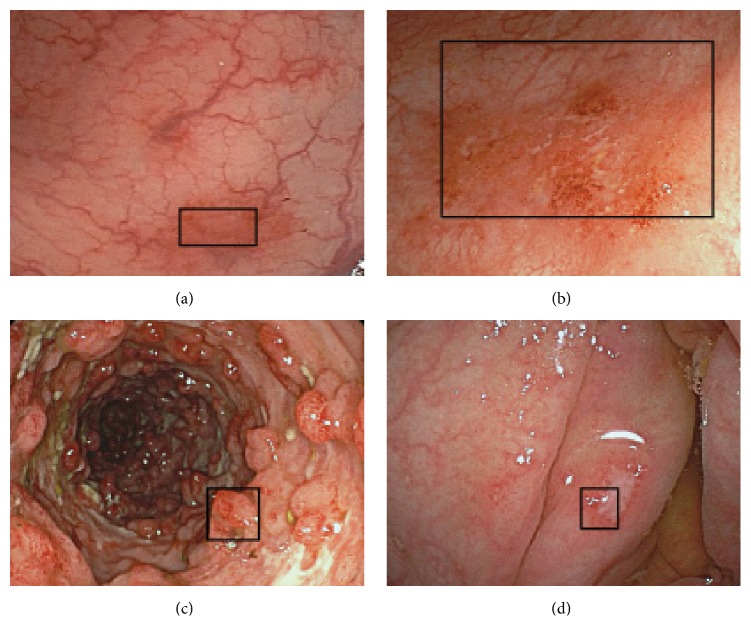
Retrieval results (black rectangles) for four actual target images.

Figure 14 presents the retrieval results for four pairs of different colonoscopy images for the same mucosal symptom in each pair, where one image was assigned as the query object with clipping and the other image was treated as the retrieval target. The results for these actual images also clearly demonstrate the feasibility of the proposed method for retrieving multiscale objects. Moreover, almost identical results were obtained for the conditions were the query and target images were reversed. In particular, the proposed method was very effective in coping with differences in sizes and locations, as shown in Figures 14(a), 14(b), and 14(c). However, for differences in terms of tilt and rotation, retrieval accuracy was poor, as shown in Figure 14(d). Naturally, this reflects the fact that the proposed method has only been developed for size and location, but, clearly, it will also be necessary to further extend the method for tilt and rotation as well for practical applications.

### 4.4. Experiment for Retrieval Performance Using Various Actual Images

**Figure 15:**
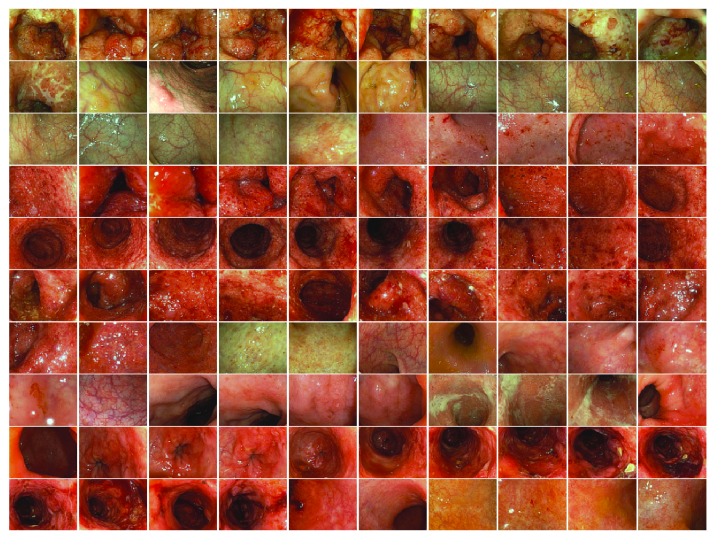
The 100 retrieval target images, which are the actual colonoscopy images.

This experiment was executed to demonstrate the retrieval performance using various actual colonoscopy images. In this experiment, 100 colonoscopy images were used as retrieval targets and four colonoscopy images selected from among them were used as the query images. These images include various degrees of UC inflammation severity (from mild, moderate, to severe), as shown in Figure 15.

**Figure 16:**
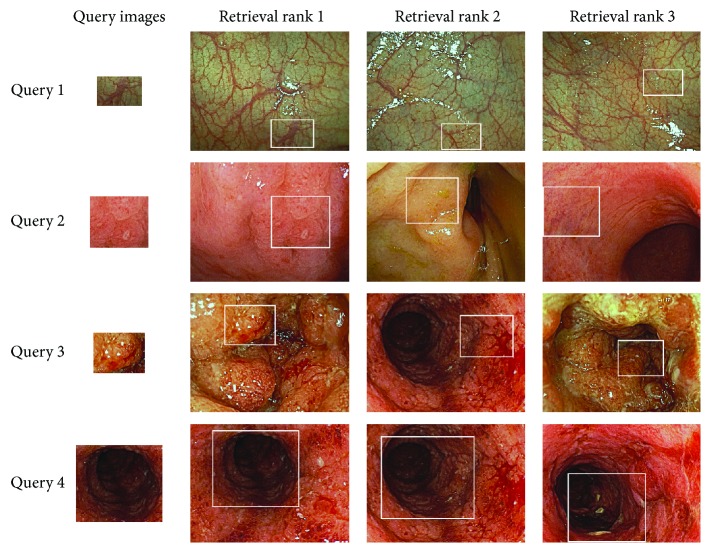
Top three retrieval image rankings for the four query images, which represent different mucosal conditions, from among the 100 target images.

Figure 16 shows the top three retrieval image rankings for the four query objects, which represent heterogeneous symptoms. For each query object, the proposed method was able to retrieve from among the 100 images ones categorized according to their similarity. In particular, the results demonstrate that the proposed method can function successfully even for severely inflamed mucosa, because the proposed integral feature can adjust for the size of the colonic mucosa within inflammations.

Table 2 presents the transitions for target images and retrieval candidates during the area selection and image selection steps. For all query objects, the initial candidates for the 100 target images exceeded 80 million candidates. However, during the area selection step, it was possible to reduce the number of retrieval candidates by at least 95%. The image selection step was also able to efficiently reduce the numbers of target images for retrieval in a stepwise fashion. These two important factors mean that the proposed method is able to reduce the computational costs, despite the major increases in computational loads incurred in extending the method to the retrieval of multiscale objects.

## 5. Conclusion

This study has proposed a method of retrieving multiscale objects from optical colonoscopy images based on HLAC. In order to retrieve objects that are similar to a target object even though based on different visible sizes, the proposed method generates tables of integral HLAC features, which are calculated by a newly extended HLAC extraction method based on an integral image technique. As the HLAC features for an arbitrarily sized clipping area can be reconfigured from the table of integral HLAC features, this approach is far more efficient than the conventional HLAC method. In the conducted verification experiments using actual colonoscopy images obtained from patients, we demonstrated that our method is capable of retrieving objects of any size and any location at high accuracy and at low computational costs and that the method is fully effective for real colonoscopy images. For the clinical use, the proposed method would be verified even more when included with clinical use, such that research into and the development of solution for diagnosis support might be advanced. We hope that the proposed method can become a key technology in developing computational support technology for optical colonoscopy diagnoses.

## Figures and Tables

**Table 1:** Experimental conditions.

Size of the target image	800 × 600 pixels
Size of the query object	Varied across the experiment
Range for the size of multiscale objects	± 10%
Degree of shift	10 pixels
Thresholds for area selection	For similarity value, less than 20 For similarity rank, higher than 10%
Threshold for 0th-order HLAC for image selection	Similarity value is less than 5
Threshold for 1st-order HLAC for image selection	Similarity value is less than 50

**Table 2:** Transitions in numbers of target images and candidates during two selections.

	Object size	Item type	Initial counts	After area selection	After image selection by 0th HLAC	After image selection by both 0th and 1st HLAC
Query 1	257 × 201	Images	100	73	68	48
		Candidates	95,760,000	4,846,155	4,834,439	3,692,044
Query 2	436 × 385	Target images	100	76	34	23
		Candidates	83,436,000	2,722,159	1,873,632	1,347,992
Query 3	293 × 257	Images	100	65	38	23
		Candidates	119,436,000	2,758,672	2,489,387	1,907,013
Query 4	225 × 147	Images	100	39	26	22
		Candidates	84,279,300	1,206,822	1,123,168	1,093,250
